# A pilot study of cystic fibrosis exacerbation response phenotypes reveals contrasting serum and sputum iron trends

**DOI:** 10.1038/s41598-021-84041-y

**Published:** 2021-03-01

**Authors:** Alex H. Gifford, Deepika Polineni, Jianghua He, Jessica L. D’Amico, Dana B. Dorman, Molly A. Williams, Amanda B. Nymon, Akshu Balwan, Theodore Budden, Jonathan B. Zuckerman

**Affiliations:** 1 Section of Pulmonary Medicine, 5C, Dartmouth-Hitchcock Medical Center, One Medical Center Drive, Lebanon, NH 03756 USA; 2 Division of Pulmonary, Critical Care and Sleep Medicine, University of Kansas Medical Center, 3901 Rainbow Boulevard, Mailstop 3007, Kansas City, KS 66160 USA; 3 Biostatistics and Data Science, University of Kansas Medical Center, Kansas City, KS USA; 4 Pulmonary and Critical Care Medicine, Maine Medical Center, Portland, ME USA; 5 Microbiology and Immunology, Geisel School of Medicine at Dartmouth, Hanover, NH USA; 6 Pulmonary and Critical Care Medicine, University of New Mexico, Albuquerque, NM USA

**Keywords:** Medical research, Signs and symptoms, Cystic fibrosis

## Abstract

The cystic fibrosis (CF) community seeks to explain heterogeneous outcomes of pulmonary exacerbation (PEX) treatment. Serum and sputum inflammatory mediators may identify people with CF (PwCF) at risk for suboptimal responses. However, lack of an established association between response phenotypes and these mediators limits clinical application. In this pilot study, we prospectively characterized treatment response phenotypes by assessing health-related quality-of-life (HRQoL) during PEX. We also measured lung function and iron-related biochemical parameters in serum and sputum. We classified subjects as sustained symptom-responders (SRs) or non-sustained symptom-responders (NSRs) based on the absence or presence, respectively, of worsened symptom scores after initial improvement. We used linear mixed models (LMMs) to determine whether trends in lung function, hematologic, serum, and sputum indices of inflammation differed between response cohorts. In 20 PwCF, we identified 10 SRs and 10 NSRs with no significant differences in lung function at PEX onset and treatment durations. SRs had better model-predicted trends in lung function than NSRs during PEX. Non-linear trends in serum and sputum iron levels significantly differed between SRs and NSRs. In adults with cystic fibrosis, PEX treatment response phenotypes may be correlated with distinctive trends in serum and sputum iron concentrations.

## Introduction

Cystic fibrosis (CF) is an autosomal recessive disease caused by mutations in *CFTR*^1^. CFTR dysfunction results in dehydrated airway secretions promoting infection, inflammation, and premature death from respiratory failure^2,3^. The clinical course of CF is typically punctuated by episodes of worsened respiratory and/or constitutional signs and symptoms called pulmonary exacerbations (PEXs). PEXs are signified by reductions in health-related quality of life (HRQoL)^4,5^, accelerated loss of lung function^6,7^, increased risk of death or lung transplant^7^, and often require costly hospitalizations^8^. In 2018, one-third of the 30,775 people with CF (PwCF) in the Cystic Fibrosis Foundation Patient Registry (CFFPR) had PEXs treated with intravenous (IV) antibiotics^9^, underscoring the prevalence of these events.

For reasons that are incompletely understood, there is marked heterogeneity to outcomes of guideline-driven PEX treatment^10^ in the U.S. CF population. Registry-based^11^, single-center^12^, and multicenter^13^ studies have shown that 15–35% of PwCF do not regain at least 90% of their baseline lung function (percent-predicted forced expiratory volume in one second, FEV1%). Because clinical practices vary among CF care centers^13–15^ and PEX treatment outcomes remain heterogeneous, there is an unmet need to characterize patient phenotypes and correlative biochemical indices that clinicians can use to guide treatment of PEXs and individualize therapies^16,17^. Trends in these indices could complement serial symptom scores in individuals with poor baseline lung function who are less likely to have significant treatment-related increases in FEV1%^13^, providing evidence of recovery that might be leveraged to reduce overtreatment.

Inflammation is a pathophysiologic hallmark of CF^18^, but limited information exists about correlations between inflammatory mediator levels and lung function^19–21^ or HRQoL^19^ during PEXs because few studies have monitored PwCF throughout a complete course of treatment. Accordingly, there remains a need to identify biochemical tests that are easily performed in clinical laboratories and can identify physiologically and symptomatically differing groups of PwCF with respect to PEX treatment response. Concentrations of certain cytokines and protein effectors of inflammation in blood and sputum from PwCF measured throughout PEXs could have prognostic utility^12,21–24^. With the exceptions of calprotectin and C-reactive protein, clinical laboratories do not routinely quantify many substances in biological samples, which is a barrier to widespread application in monitoring treatment responses^25,26^.

Interleukin-6 (IL-6), a pro-inflammatory cytokine found at high concentrations in blood and sputum from PwCF^27^, stimulates the liver to produce hepcidin-25^28^, a hormone that reduces blood iron levels by attenuating gastrointestinal iron absorption^29^ and triggering mononuclear cells to sequester iron^30^. In adults with CF, we found that serum iron levels were lower and sputum iron, serum IL-6, and serum hepcidin-25 levels were higher immediately before PEX treatment^31^. This pattern is consistent with the mechanisms by which IL-6 and hepcidin-25 link iron homeostasis to inflammation^32^. A limitation of our previous work^31^ was the omission of serial assessments of lung function and HRQoL to compare to biomarkers of iron homeostasis.

The current study had a master aim of phenotyping cohorts of adults with CF with respect to HRQoL, FEV1% and other clinical metrics, along with laboratory indices of iron homeostasis measured at pre-specified points throughout PEX treatment. Anticipating that our study participants would respond heterogeneously to PEX treatment, consistent with common clinical experience and published reports^11–13^, we sought to test a primary hypothesis that this diversity would be reflected in HRQoL scores and measures of lung function. We also sought to test a secondary hypothesis that biomarkers of iron homeostasis would likewise indicate the relative salutary effects of PEX treatment on the inflammatory state over time.

## Methods

### Study subjects and design

We conducted a prospective observational study of adults with CF as per diagnostic standards^33^. Subjects were hospitalized for PEX treatment at Dartmouth-Hitchcock Medical Center (DHMC) or Maine Medical Center (MMC) between September 2014 and July 2017. Attending pulmonologists (JBZ, AHG) made PEX treatment decisions including: interruption or continuation of chronic medications^34,35^ and selection plus duration of IV antibiotics. Subjects completed IV antibiotic courses at home or inpatient at the pulmonologist’s discretion, but all returned to clinic for their end of treatment study assessments.

At the time of enrollment, baseline characteristics of subjects were recorded (Table S1), and subjects were followed until they experienced a PEX, at which point they were included in the study analysis. We used the Akron Pulmonary Exacerbation Score (PES) to define PEX onset. The Akron PES is a clinical tool designed to standardize the use of antibiotics to treat CF PEXs and is used in clinical practice, including by the adult CF programs of both study centers. The instrument classifies data collected during routine office visits into three domains (systemic signs and symptoms, pulmonary signs and symptoms, and objective measurements). The Akron PES ranges from 0 to 30 with a score ≥ 5 signaling PEX onset^36^. Subject data was analyzed from PEX onset through the end of treatment with IV antibiotics.

### Subject self-assessment of HRQoL

We used the CF Respiratory Symptom Diary (CFRSD), licensed by the Seattle Quality of Life Group at the University of Washington, to obtain HRQoL data. The CFRSD is a validated eight-question, self-completed instrument that evaluates the symptom burden of PwCF during the preceding 24-h epoch^37,38^. We summed raw CFRSD responses and calculated the Chronic Respiratory Infection Symptom Score (CRISS) using a conversion table^39^. The CFRSD-CRISS ranges from 0 to 100 with higher scores indicating higher symptom burden.

### Definitions of sustained and non-sustained symptom responder cohorts

We classified subjects whose CFRSD-CRISS steadily declined between onset of PEX and the end of treatment as sustained symptom-responders (SRs). We classified subjects whose CFRSD-CRISS initially declined but increased before the end of treatment as non-sustained symptom-responders (NSRs).

### Diagnostic testing

We used clinical autoanalyzers to measure complete blood counts, serum iron, transferrin saturation (TSAT), and total iron binding capacity (TIBC). The clinical microbiology laboratory at each site performed sputum cultures. We assayed spontaneously expectorated sputum samples for inorganic iron using inductively coupled plasma-mass spectrometry^40^. Spirometry was performed per ATS/ERS standards^41^. We measured serum IL-6 levels in triplicate using solid phase sandwich enzyme-linked immunosorbent assay (ELISA) kits (Human Quantikine; R&D Systems; Minneapolis, MN) with a detection range of 3.1–300 pg/ml. Serum hepcidin-25 was measured by competitive ELISA (Intrinsic LifeSciences, LLC; La Jolla, CA) using published methods^42^. The lower limits of quantitation and detection for the serum hepcidin-25 assay were 19 ng/ml and 5 ng/ml, respectively.

### Statistical analyses

Median values of continuous variables were compared between cohorts using the Wilcoxon rank sum test, and categorical variables were compared by Chi-square tests. Kaplan–Meier analysis was used to compare durations of PEX treatment. Linear mixed models (LMMs) were used to test whether the trajectory of each outcome differed by cohort and accommodate missing data in this multiyear study. Variables with right-skewed distributions were natural log-transformed before inclusion in LMMs. Quadratic and linear terms for time after PEX onset and interactions between these time variables and cohort designation were tested in a hierarchical order: quadratic interaction, linear interaction, quadratic time term, and linear time term. The quadratic term for time was used to allow a non-linear time trend, and no higher order terms such as the cubic term were considered due to the small sample size. If the quadratic interaction term was significant or borderline ((p < 0.05); and 0.05 < p < 0.1, respectively), the full model was kept. Otherwise, the quadratic interaction term was removed and the significance of the linear interaction term was examined. Consistent with analytic methods in hypothesis-generating studies, this process was stopped when a significant or borderline result was found or when all variables were removed from the model. All LMMs included a constant term to adjust for between-group differences in the outcome of interest at PEX onset. A final model that retained the quadratic and/or linear interaction term(s) suggested a potential difference of time trend between cohorts for the outcome of interest. Plots of the estimated mean trajectories with point-wise confidence intervals illustrated between-cohort differences in time trend. LMMs were also used to examine associations between the change in each physiologic or biochemical index with the change in CFRSD-CRISS during PEX treatment. In these models, the outcome was the repeated measures of CFRSD-CRISS after PEX onset, and the predictor was the change in each index from day 0 and CFRSD-CRISS at day 0, which was included to control for the effect of regression to the mean. Due to the small sample size, no control for multiple tests was applied, and because this study was hypothesis-generating, borderline-significant terms were retained in the final models.

### Ethics approval and consent to participate

The study protocol was approved by the Committee for the Protection of Human Subjects (CPHS) at Dartmouth College (#28100) and the Institutional Review Board for Maine Medical Center (#4700). All experimental protocols involving human data and/or biological samples were conducted in accordance with the Declaration of Helsinki. Written informed consent was obtained from study participants.

## Results

### Characteristics of study subjects

**Figure 1:**
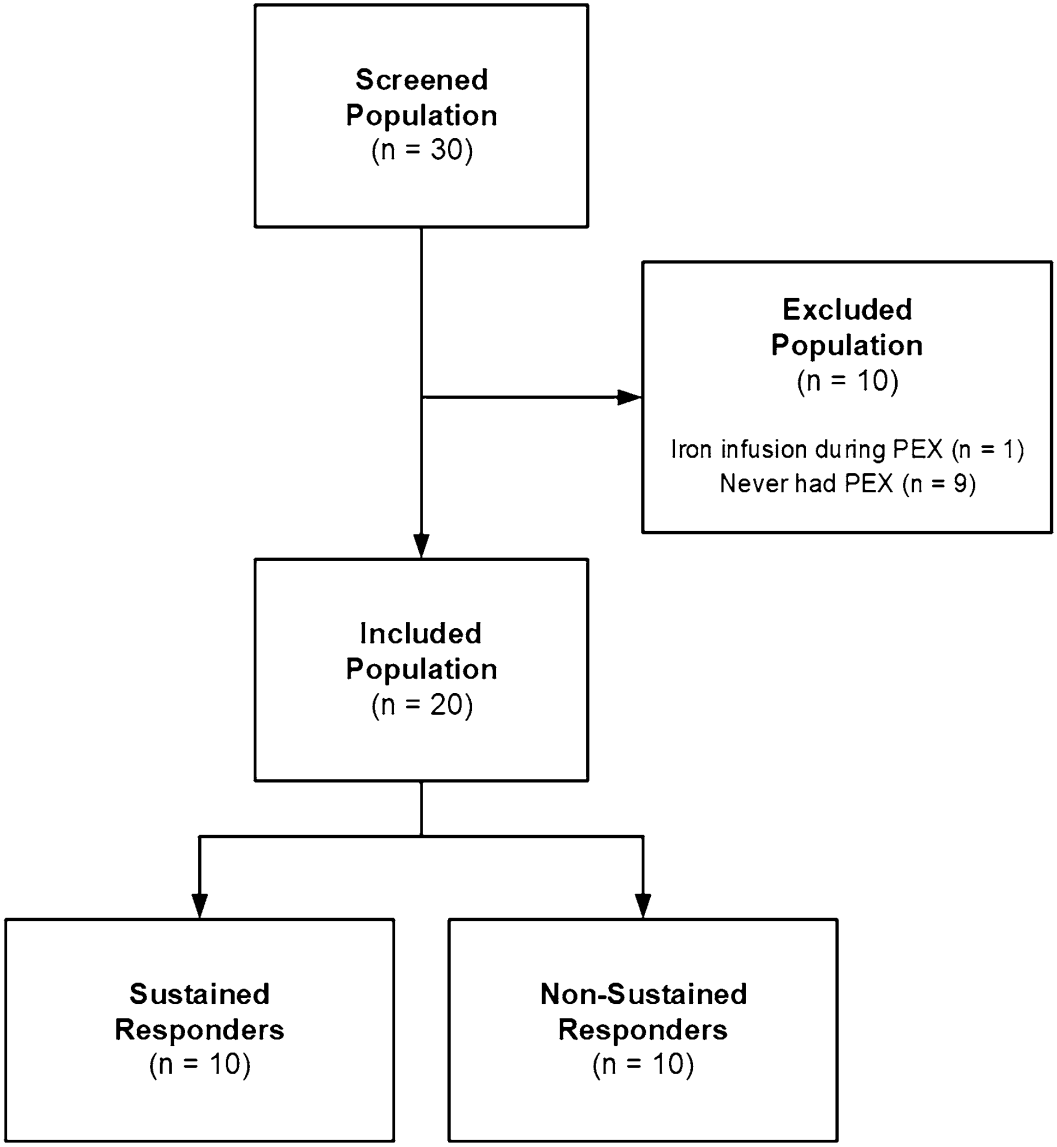
STROBE diagram showing subject flow during the study.

We screened 30 subjects, and analyzed data from 20 subjects that had protocol-defined PEXs (Fig. 1). We excluded 9 subjects who did not meet PEX criteria and 1 subject who received IV iron transfusion (Fig. 1). Two-thirds of included subjects were enrolled at DHMC, with the remaining subjects enrolled at MMC. There were equal numbers of males and females. In general, subjects were older adults with severe lung disease (Table 1). Sixty percent were homozygous for the F508del-*CFTR* mutation; all but one of the remaining subjects were F508del heterozygotes. Fifty percent of subjects had *Pseudomonas aeruginosa* identified on sputum culture (n = 6 NSR; n = 4 SR; p = 0.66). One subject was chronically taking a highly effective CFTR modulator treatment, ivacaftor, and two subjects were taking lumacaftor/ivacaftor modulator therapy; all subjects chronically expectorated sputum. Clinical characteristics of the included participants, including baseline spirometry at study enrollment, are presented in Table S1.

**Table 1:** Baseline clinical characteristics of the study population prior to PEX onset.

Subject	Response cohort	Age (years)	Sex	F508del-CFTR × 2 (yes/no)	FEV1, L (% pred)	FVC, L (% pred)	Weight (kg)	Akron PES	CFRSD-CRISS	Sputum culture	Chronic medications
1	SR	27	F	Yes	2.85 (91)	3.70 (101)	56.8	1	23	AX	DA, HS, COL, ICS, AZTH
2	SR	27	M	Yes	2.42 (50)	4.62 (78)	69.9	0	23	MSSA	DA, HS, ICS, TMP-SMX
3	SR	25	M	Yes	4.00 (78)	5.53 (88)	99.6	4	34	PsA	DA, AZTH, ICS, COL
4	SR	24	F	Yes	2.39 (79)	3.56 (102)	49.9	0	29	MSSA	DA, HS, TOB, AZTH
5	NSR	20	M	No	1.35 (36)	1.91 (44)	48.2	3	34	AX	DA, HS, TOB, ICS, IVA
6	NSR	38	F	Yes	0.56 (23)	1.22 (41)	44.0	4	52	MRSA, PsA	DA, HS, TOB, AZTH, PRED
7	NSR	30	M	No	0.90 (22)	1.77 (36)	65.1	0	34	PsA	DA, HS, AZTH, COL
8	SR	32	M	Yes	2.84 (77)	4.08 (91)	66.2	1	23	BC	DA, HS, COL, TMP-SMX
9	NSR	20	F	No	1.65 (50)	2.52 (67)	50.8	0	41	BG	DA, HS
10	NSR	25	M	Yes	3.21 (70)	4.56 (83)	60.5	1	41	MRSA, PsA	DA, HS, AZ
11	SR	46	M	No	0.96 (25)	1.75 (36)	70.6	3	41	MRSA, PsA	DA, HS, AZTH, COL, DOX
12	SR	33	F	No	0.96 (34)	1.42 (42)	43.1	2	29	MRSA, PsA	DA, HS, AZTH, COL, TOB
13	NSR	27	F	Yes	1.96 (64)	3.05 (86)	56.9	0	23	BM, HI	DA, TOB, AZTH, LUM/IVA
14	SR	35	F	No	1.42 (46)	2.45 (65)	57.6	1	37	MSSA, PsA	HS, AZ, AZTH
15	SR	27	F	Yes	1.39 (39)	2.77 (66)	57.8	3	44	MSSA, BV	DA, AZ
16	NSR	34	M	Yes	1.08 (26)	2.34 (46)	72.1	0	37	MSSA	DA, HS, AZ, LUM/IVA
17	SR	20	F	No	2.14 (71)	2.88 (85)	47.7	0	34	MRSA	DA, HS, ICS
18	NSR	35	M	Yes	1.92 (41)	2.58 (45)	59.9	4	52	PsA, MSSA	DA
19	NSR	22	M	Yes	1.07 (24)	1.98 (37)	71.5	1	37	PsA, MSSA	DA, HS, ICS, AZ
20	NSR	52	F	No	1.06 (35)	1.70 (44)	74.0	0	49	PsA	DA, HS, AZ, PRED
Mean (SD)		29.9 (8.5)			1.80 (0.9) 49 (22)	2.8 (1.2) 64 (23)	61.1 (13.3)	1.4 (1.5)	35.9 (9.2)		

F508del × 2 = F508del-*CFTR* homozygote; PES = Pulmonary Exacerbation Score; CFRSD-CRISS = CF Respiratory Symptom Diary-Chronic Respiratory Infection Symptom Score; AX = *Achromobacter xylosoxidans*; MSSA = methicillin-sensitive *Staphylococcus aureus*; MRSA = methicillin-resistant *Staphylococcus aureus*; PsA = *Pseudomonas aeruginosa*; BC = *Burkholderia cepacia*; BM = *Burkholderia gladioli*; BM = *Burkholderia multivorans*; BV = *Burkholderia vietnamiensis*; HI = *Haemophilus influenzae*; DA = dornase alfa; HS = nebulized hypertonic saline; AZ = aztreonam lysine for inhalation; TOB = inhaled tobramycin; COL = inhaled colistin; AZTH = azithromycin; ICS = inhaled corticosteroid; PRED = prednisone; IVA = ivacaftor (CFTR modulator); LUM/IVA = lumacaftor/ivacaftor (CFTR modulator); TMP-SMX = trimethoprim-sulfamethoxazole; DOX = doxycycline.

### Identification of two cohorts with distinct symptomatic responses to PEX treatment

**Figure 2:**
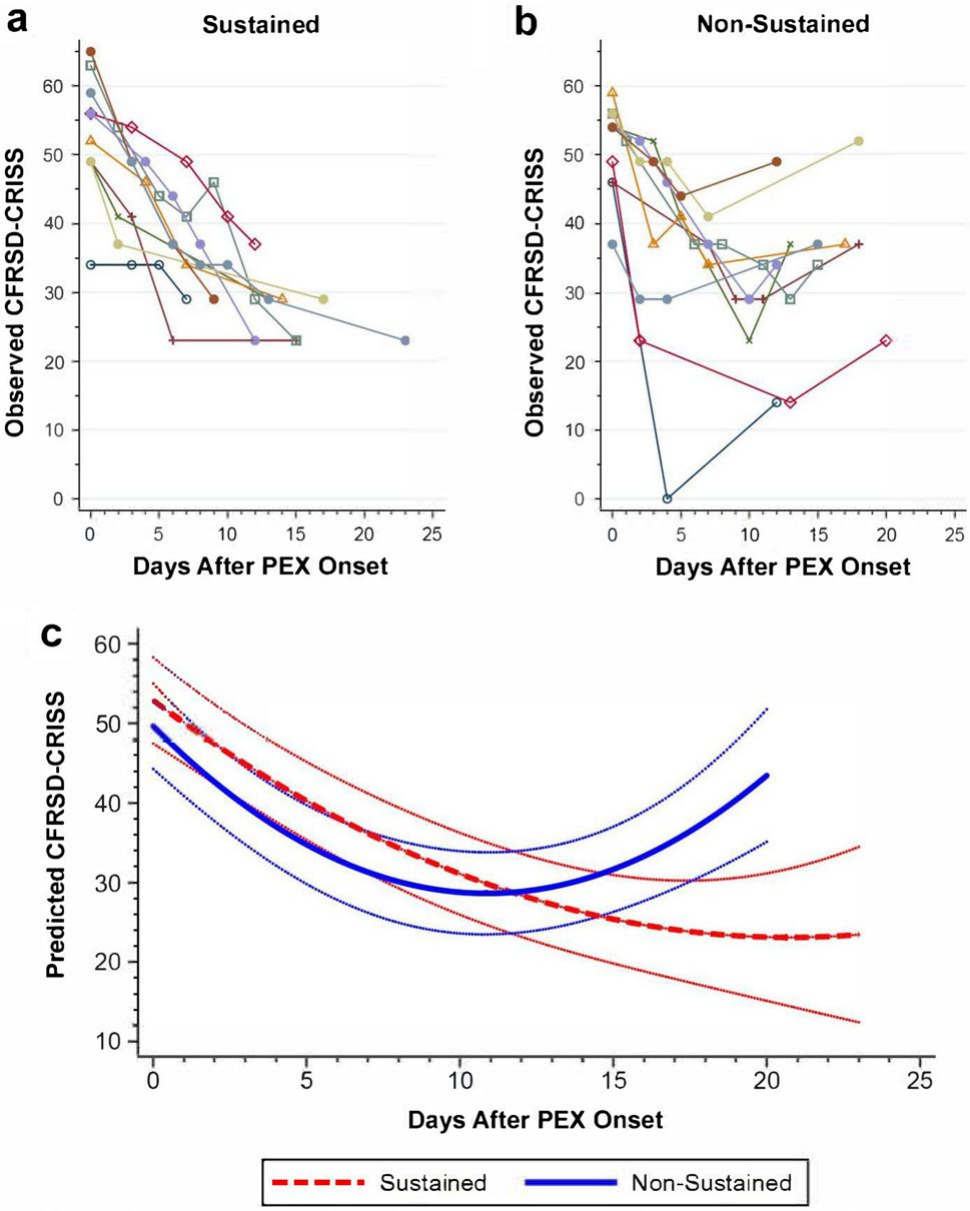
Trends in CFRSD-CRISS during PEX treatment. “Spaghetti plots” of raw CFRSD-CRISS data identified a cohort with a sustained decline in symptom burden (**a**) and a cohort with an initial decline and terminal rebound in symptom burden (**b**). These distinctions were modeled using a linear mixed model (**c**), adjusting estimates for any difference in CFRSD-CRISS between sustained and non-sustained symptom responders at PEX onset. Curves around each estimated trend reflect 95% C.I. of parameter estimates.

Inspection of raw within-subject CFRSD-CRISS data in the form of “spaghetti plots” revealed two discrete patterns of variation during PEX treatment. Ten subjects had a steady decline in CFRSD-CRISS (Fig. 2a) characterizing them as SRs; and ten subjects had an initial decline and terminal rebound in CFRSD-CRISS making them NSRs (Fig. 2b). Median CFRSD-CRISS at PEX onset were not statistically different between groups. The LMM for CFRSD-CRISS after PEX onset as a function of time showed a highly significant interaction between cohort and the quadratic time term, implying different time trends between cohorts (Table S2). The estimated mean trajectories of CFRSD-CRISS scores and 95% confidence intervals (CIs) are shown (Fig. 2c). These trends are consistent with the observed raw data (Fig. 2b). Kaplan–Meier curves based on inpatient length of stay (Fig. S1) showed that median treatment durations for SRs and NSRs were not statistically different (13.5 vs 15.0 days, respectively; p = 0.54).

### FEV1% trends differed between SRs and NSRs

**Figure 3:**
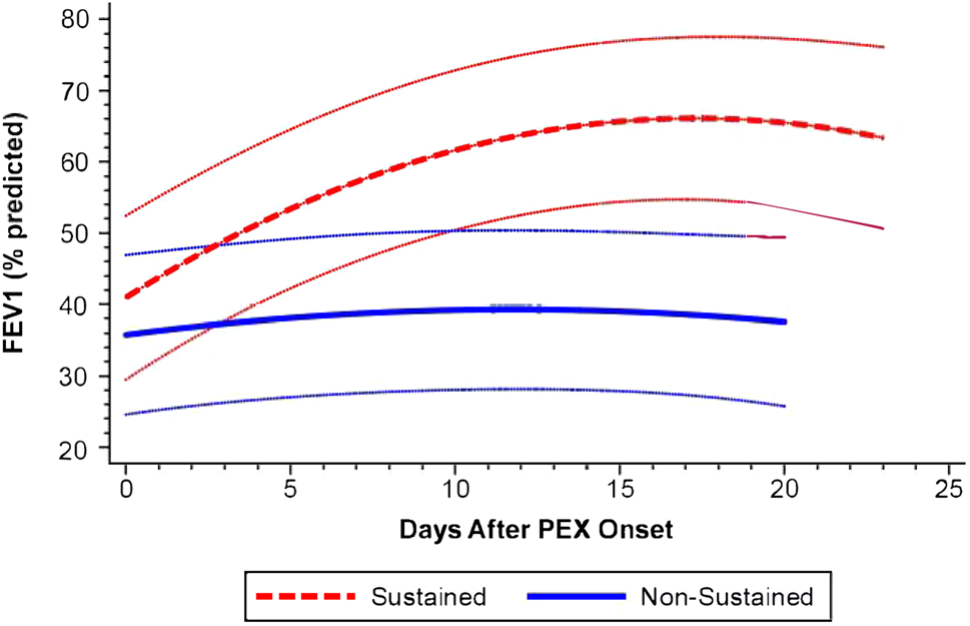
Model-predicted trends in FEV1% during PEX treatment for sustained and non-sustained symptom responders. Curves around each estimated trend reflect 95% C.I. of parameter estimates.

At the time of PEX onset, there was no significant difference in FEV1% between SRs and NSRs (39.6 ± 9.0% vs. 37.3 ± 5.2%, p = 0.94). However, not all subjects completed spirometry at PEX onset (n = 5 SR and n = 2 NSR with missing FEV1%). The LMM for FEV1% suggested different time trends between SRs and NSRs with a significant interaction between cohort and quadratic time terms (p = 0.02, Table S3); estimated trajectories and 95% CIs are shown (Fig. 3). The FEV1% trend for SRs increased shortly after treatment initiation and plateaued nearing treatment completion, whereas this trend was almost flat for NSRs.

### Serum IL-6 and hepcidin-25 levels trended lower in SRs and NSRs

Log transformation was applied to serum hepcidin-25 and serum IL-6 data for right-skew. LMMs for both variables had an interaction between cohort and linear time term with borderline-significant p-values (0.05 < p < 0.1). Quadratic interaction terms were dropped, as p-values were > 0.1) (Tables S4 and S5). Thus, the analytic approach did not show statistically significant differences in serum IL-6 and hepcidin-25 trajectories between SRs and NSRs.

### Serum and sputum iron trends differed between SRs and NSRs

**Figure 4:**
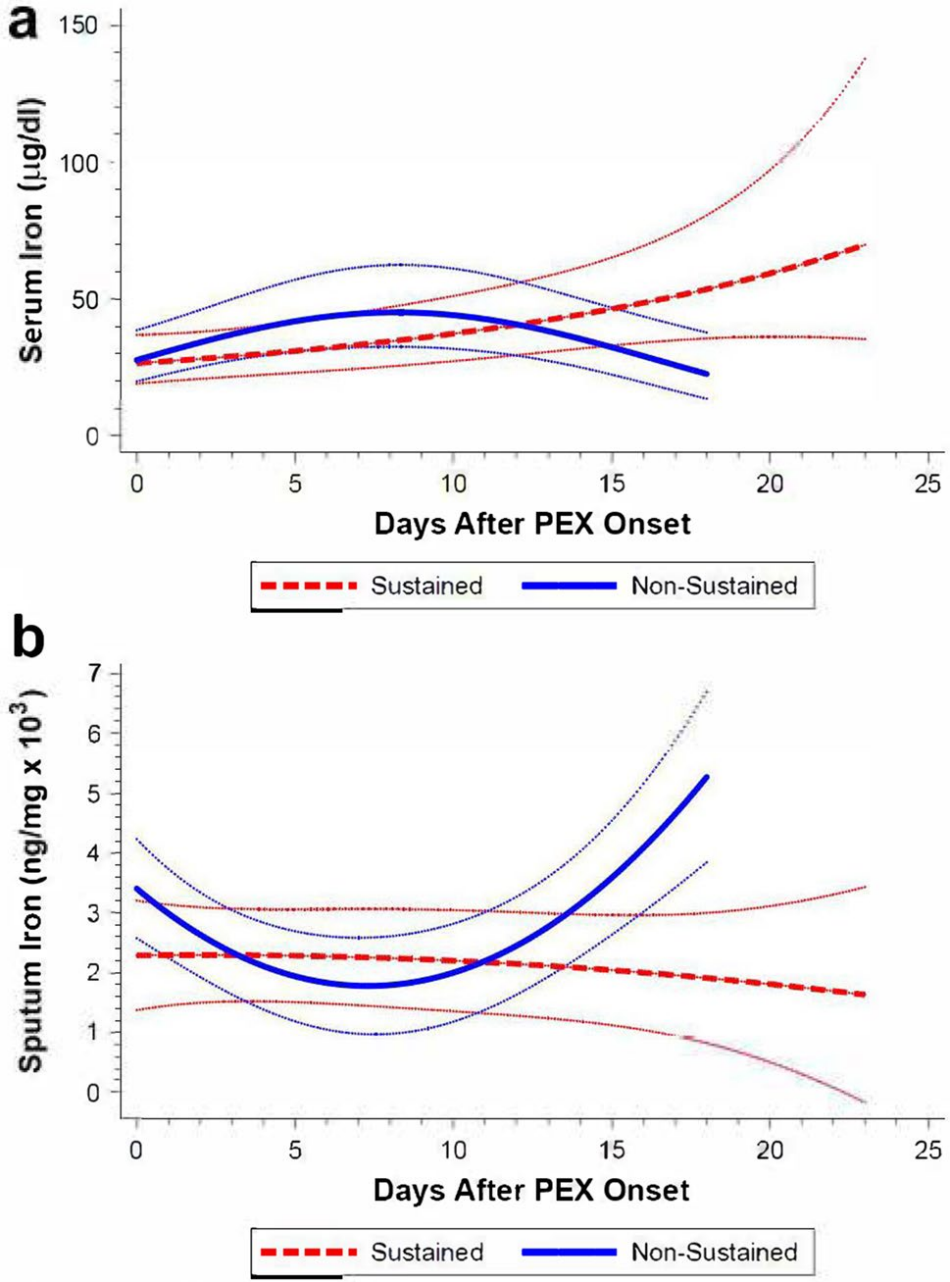
Model-predicted trends in serum (**a**) and sputum (**b**) iron concentrations for sustained and non-sustained symptom response cohorts during PEX treatment.

Log transformation was also applied to serum iron and sputum iron data for right-skew. The LMMs for both serum and sputum iron showed a significant interaction of cohort and quadratic time term (Tables S6 and S7), again suggesting different time trends for serum and sputum iron between groups. The estimated mean trajectories and 95% CIs for both variables using the original units of measure scale are shown (Fig. 4). For SRs, the estimated mean of serum iron increased over time and that of sputum iron was relatively stable (Fig. 4a,b). For NSRs, the estimated means of serum iron initially increased but then decreased (Fig. 4a), while that of sputum iron first decreased and then increased around day 7 of treatment (Fig. 4b).

Analysis of the association between serum and sputum iron changes in all subjects showed a modest negative correlation (r = − 0.34, p = 0.02; Fig. S2).

### Summary of findings of changes in additional variables during PEX treatment

LMMs were applied to additional hematologic tests to identify trend differences between cohorts (Table S8). LMMs were also used to explore whether changes in relevant physiologic and/or biochemical indices were related to changes in CFRSD-CRISS (Table S9). Notably, total white blood count (WBC) trends did not differ between cohorts. However, in all subjects, analyses identified negative associations for changes in FEV1%, serum iron, TSAT, TIBC, and percent lymphocytes (WBC %L) with changes in CFRSD-CRISS. Positive associations were observed for changes in CFRSD-CRISS and percent neutrophils (WBC %N), serum IL-6, and serum hepcidin-25 in all subjects (Table S9).

## Discussion

This pilot study characterized the evolution of respiratory symptom burden, lung function, and biochemical indices of inflammation and iron homeostasis in adults with CF during PEX treatment. We found that HRQoL (CFRSD-CRISS) and lung function (FEV1%) trends (Figs. 2 and 3) differed significantly between two equally sized cohorts over courses of similar treatment duration (Fig. S1). These findings supported our primary hypothesis that treatment responses significantly differ in PwCF and defined PEX treatment response phenotypes in clinically-meaningful terms (SRs and NSRs). Although these SR and NSR groups could not be distinguished by unique trends in serum IL-6 and hepcidin-25 levels (Tables S4 and S5), trends in serum and sputum iron (Fig. 4a,b, Tables S6, S7) were significantly divergent between cohorts. We also found that changes in hematologic markers of inflammation (WBC %N, serum IL-6, and serum hepcidin-25) correlated positively with treatment-related change in CFRSD-CRISS while changes in FEV1%, serum iron, TSAT, TIBC and WBC %L correlated negatively with treatment-related changes in CFRSD-CRISS (Table S9). These associations suggest that iron deficiency in PwCF, as reflected by lower serum iron levels, may be a dynamic and relative state induced by IL-6 and hepcidin-25 during PEXs.

The association between unfavorable clinical outcomes of PEX treatment and synchronous elevations in sputum iron and reductions in serum iron has not heretofore been reported. Data from this study and our previous work^31^ suggest that CF PEXs are characterized by alterations in iron homeostasis. This observation is congruent with in vitro studies in bronchial epithelial co-culture models of CF lung infection that have identified a link between transepithelial iron shifts into airway surface liquid and increased apical biofilm growth of *Pseudomonas aeruginosa* in an F508del isogenic cell line^43^. Findings from Hunter et al.^44^ additionally back the concept that excess iron in the lungs of PwCF is deleterious, as higher sputum ferrous iron levels correlated with lower FEV1%. We now question whether suboptimal CF PEX treatment responses are associated with net iron transfer from the bloodstream into the airways. This idea is supported by our finding of a negative correlation between Δserum iron and Δsputum iron during PEX treatment (Fig. S2). However, if this phenomenon occurs during CF PEXs, our pilot data suggest that it is not closely linked to changes in serum IL-6 (Table S4) and hepcidin-25 (Table S5).

Mechanisms by which iron accumulates in the lungs^45^ and sputum^31,44,46–49^ of PwCF remain enigmatic. CF sputum iron content has been shown to correlate positively with *Pseudomonas aeruginosa* colony forming units (CFUs)^49^, suggesting that iron levels are proportional to the quantitative load of bacteria in the CF airway. However, positive correlations of sputum iron with sputum cytokine concentrations and cell counts also implicate other host factors in airway microbiota homeostasis^49^. Because we only measured total inorganic iron in sputum, we cannot add to the literature supporting one or both of these possibilities. Nonetheless, our findings support the clinical relevance of excess iron in the lungs of PwCF.

We acknowledge several additional limitations of this work. First, we studied a small sample of twenty subjects (Table 1) in this rare disease and conducted multiple analyses. Findings from this investigation, while provocative, should be considered exploratory, and should be validated in a larger study. A larger sample size and more frequent biospecimen assessment could better determine the minimum number and optimal collection time points to pragmatically predict differential treatment responses. Performance of testing panels, rather than assays for one or a few markers, may ultimately be necessary to characterize the range of PEX responses in diverse populations. Second, these results from two CF centers may limit the generalizability of our observations. However, our subjects did have clinical attributes similar to those of adults with CF during PEXs in larger studies^19,50,51^. The LMM coefficient for ΔCFRSD-CRISS as a function of ΔFEV1% (− 0.70, p < 0.001) (Table S9) is similar in magnitude to that reported in the large multicenter Standardized Treatment of Pulmonary Exacerbations (STOP) study (n = 173)^52^.

Third, CF Center norms related to the diagnosis and treatment of PEXs could have influenced our results. We tried to address this issue by using the Akron PES to define PEX onset at both locations (Table 1). While we concede that different diagnostic criteria have been used to define PEXs in CF research^53^, we have previously used the Akron PES in a study of adults with CF^54^.The Akron PES was also designed to standardize antibiotic prescription in CF PEX^36^. Based on the observational nature of this study using standard of care therapy, we did not restrict treatment selection or duration. It is unclear whether an interventional study controlling these factors would yield similar results. However, a strength of our approach was that it tested our hypotheses under real-world conditions.

Notably, baseline characteristics at enrollment showed that all SRs had lower FEV1% than NSRs, but there were no significant differences in serum iron levels or other clinical and biochemical parameters (Table S1). Differences in baseline FEV1% raise the possibility that PwCF with worse lung disease may be more prone to having suboptimal symptoms responses during treatment. At the onset of PEX, PwCF frequently cannot perform spirometry for a variety of reasons (e.g., pain, fatigue, hemoptysis). One limitation of our study is missingness of complete FEV1% data at PEX onset, which may lead to Type II error for the comparison of FEV1% between cohorts at PEX onset. Nevertheless, these pilot findings of novel exacerbation symptom response phenotypes in association with serum iron, or FEV1, warrant further validation. Our findings support the importance of acute variation of readily accessible serum analytes over the course of CF PEX, thereby highlighting their potential utility to objectively distinguish and predict response phenotypes in diverse populations.

## Conclusions

In summary, we have shown in this prospective, two-center, observational pilot study of twenty adults with CF who were hospitalized for PEXs that iron homeostasis differed between exacerbation treatment phenotypes defined by symptomatic and spirometric responses to treatment with IV antibiotics. Analyses of serum iron, TSAT, and TIBC, all of which are easily performed by clinical laboratories, may prove to be pragmatic and readily adopted tools for treatment teams. Sputum iron assays may prove to be less practical in light of the complexity of the test and the observation that with increasing use of highly effective CFTR modulator therapies, many patients no longer expectorate copious sputum. Larger studies to validate HRQoL phenotypes in CF PEXs and correlative analyses of inflammatory and/or iron indices are warranted. Future clinical trials may be informed by defining exacerbation phenotypes and trends in serum and sputum iron, particularly as novel disruptors of microbial iron acquisition and utilization are under investigation as anti-infective therapies in CF^55,56^.

## Supplementary information

Supplementary information.

## Abbreviations

CF: Cystic fibrosis
CFFPR: Cystic Fibrosis Foundation Patient Registry
CFRD: Cystic fibrosis related diabetes
CFRSD: Cystic Fibrosis Respiratory Symptom Diary
CFTR: Cystic fibrosis transmembrane conductance regulator
CI: Confidence interval
CRISS: Chronic Respiratory Infection Symptom Score
DHMC: Dartmouth-Hitchcock Medical Center
ELISA: Enzyme-linked immunosorbent assay
FEV1: Forced expiratory volume in one second
FEV1%: Percent predicted forced expiratory volume in one second
HRQoL: Health related quality of life
IL-6: Interleukin-6
IV: Intravenous
LMM: Linear mixed model
MCHC: Mean corpuscular hemoglobin concentration
MCV: Mean corpuscular volume
MPV: Mean platelet volume
MMC: Maine Medical Center
NSR: Non-sustained symptom responder
PES: Pulmonary exacerbation score
PEX: Pulmonary exacerbation
PwCF: People with cystic fibrosis
RDWCV: Coefficient of variation of the red cell distribution width
RDWSD: Standard deviation of the red cell distribution width
SD: Standard deviation
SR: Sustained symptom responder
TIBC: Total iron binding capacity
TSAT: Transferrin saturation
WBC: White blood cell
WBC %L: Percentage lymphocytes on white blood cell differential
WBC %N: Percentage neutrophils on white blood cell differential
