# Supplementary material for: A pilot study of cystic fibrosis exacerbation response phenotypes reveals contrasting serum and sputum iron trends
Source: Sci Rep. 2021 Mar 1;11:4897. doi: 10.1038/s41598-021-84041-y (PMC7921142; doi:10.1038/s41598-021-84041-y)
Supplement: Supplementary file 1 — Supplementary information. [file 41598_2021_84041_MOESM1_ESM.docx]

**A PILOT STUDY OF CYSTIC FIBROSIS EXACERBATION RESPONSE PHENOTYPES REVEALS CONTRASTING SERUM AND SPUTUM IRON TRENDS**

Alex H. Gifford, M.D.^1^ **¶**; Deepika Polineni, M.D., M.P.H.^2^ **¶**; Jianghua He, Ph.D.^3^

Jessica L. D’Amico, D.O.^4^; Dana B. Dorman, R.N., M.S.N.^1^; Molly A. Williams, B.S.^1^;

Amanda B. Nymon, B.S.^5^; Akshu Balwan, M.D., M.B.B.S.^6^; Theodore Budden, B.S.^2^;

Jonathan B. Zuckerman, M.D.^4^

**AFFILIATIONS**:

1. Pulmonary Medicine, Dartmouth-Hitchcock Medical Center, Lebanon, NH
2. Pulmonary and Critical Care Medicine, University of Kansas Medical Center, Kansas City, KS
3. Biostatistics and Data Science, University of Kansas Medical Center, Kansas City, KS
4. Pulmonary and Critical Care Medicine, Maine Medical Center, Portland, ME
5. Microbiology and Immunology, Geisel School of Medicine at Dartmouth, Hanover, NH
6. Pulmonary and Critical Care Medicine, MaineGeneral Medical Center, Augusta, ME

**¶** Authors contributed equally

**CORRESPONDING AUTHORS**:

Alex H. Gifford, M.D.

Section of Pulmonary Medicine, 5C

Dartmouth-Hitchcock Medical Center

One Medical Center Drive

Lebanon, NH 03756

E-mail: [alex.h.gifford@hitchcock.org](mailto:alex.h.gifford@hitchcock.org)

Telephone: (603) 650-5533

FAX: (603) 650-0580

Deepika Polineni, M.D., M.P.H.

Division of Pulmonary and Critical Care Medicine

University of Kansas Medical Center

3901 Rainbow Boulevard, Mailstop 3007

Kansas City, KS 66160

E-mail: [dpolineni@kumc.edu](mailto:dpolineni@kumc.edu)

Telephone: (913) 588-6046

FAX: (913) 588-4098

**Supporting Information**

|  |  |  |  |
| --- | --- | --- | --- |
|  | **Subjects without PEX**  **(n = 10)** | **Sustained**  **Responders**  **(n = 10)** | **Non-Sustained Responders**  **(n = 10)** |
|  |  |  |  |
|  |  |  |  |
| Age, yrs | 33.5 (19-58) | 31 (25-46) | 26 (21-52) |
| Male / Female, n | 5 / 5 | 7 / 3 | 6 / 4 |
| Body weight, kg | 59.3 (52.4-78.6) | 57.7 (43.1-99.6) | 60.2 (44.0-74.0) |
| F508del-CFTR x 2, n (%) | 5 (50) | 6 (60) | 6 (60) |
| FEV1, % predicted | 69.0 (22-97) | 60.5 (25-91) | 35.5 (22-70) *^†^ |
| FVC%, % predicted | 77.5 (37-96) | 81.5 (36-102) | 44.5 (36-86) |
| MRSA, n (%) | 0 (0) | 3 (30) | 2 (20) |
| *Burkholderia* species, n (%) | 0 (0) | 2 (20) | 2 (20) |
| *P. aeruginosa*, n (%) | 8 (80) | 4 (40) | 6 (60) |
| Akron PES | 2 (0-4) | 0.5 (0-4) | 1.0 (0-4) |
| CFRSD-CRISS | 36.5 (23-48) | 31.5 (23-44) | 39 (23-52) |
| WBC, x10^3^/μl | 9.3 (6.7-17.2) | 9.2 (5.0-14.2) | 11.0 (4.5-15.5) |
| WBC neutrophils, % | 73.5 (53.0-86.0) | 68.8 (54.3-75.1) | 77.2 (56.1-81.4) |
| Hemoglobin, gm/dl | 12.9 (12.0-15.8) | 12.6 (9.7-15.1) | 13.0 (11.8-16.5) |
| Platelets, x10^3^/μl | 333 (165-422) | 333 (195-551) | 296 (207-374) |
| Serum iron, μg/dl | 59.5 (34-108) | 44.5 (10-160) | 27.5 (17-80) |
| Sputum iron, ng/mg | N/A | 2,415 (373-10,366) | 1,711 (677-8,352) |
| Serum hepcidin-25, ng/ml | N/A | 52.9 (10.5-214.3) | 64.6 (9.4-165.9) |
| Serum IL-6, pg/ml | N/A | 11.6 (1.6-22.8) | 9.4 (2.6-17.3) |
|  |  |  |  |

**Table S1**. Baseline clinical and biochemical characteristics of subjects who were screened but never experienced a PEX, SRs, and NSRs. N/A = not available. Data are presented as median (range) unless otherwise noted. * p < 0.05 vs. SRs. ^†^ p < 0.05 vs. subjects without PEX.

| **Model Parameter** | **Coefficient (β)** | **95% C.I. (β)** | **p-value** |
| --- | --- | --- | --- |
|  |  |  |  |
| Quadratic Interaction term (Group • days^2^) | 0.11 | 0.04, 0.18 | 0.003 |
| Linear Interaction term (Group • days) | 0.09 | -0.55, 0.73 | 0.78 |
| Quadratic Time After PEX Onset (days^2^) | 0.07 | 0.02, 0.12 | 0.004 |
| Linear Time After PEX Onset (days) | -2.2 | -2.6, -1.7 | <0.0001 |
| Difference in CFRSD-CRISS at PEX Onset (NSR - SR) | -5.5 | -12.5, 1.5 | 0.12 |
| Constant (CFRSD-CRISS) | 40.3 | 35.4, 45.2 | <0.0001 |
|  |  |  |  |

**Table S2**. Linear mixed model (LMM) describing CFRSD-CRISS over time between two PEX treatment response groups, sustained responders (SRs, n = 10) and non-sustained responders (NSRs, n = 10). C.I. = confidence interval. Number of observations = 96. Number of subjects = 20. Observations per group: minimum = 3, maximum = 7, average = 4.8.

| **Model Parameter** | **Coefficient (β)** | **95% C.I. (β)** | **p-value** |
| --- | --- | --- | --- |
|  |  |  |  |
| Quadratic Interaction term (Group • days^2^) | 0.06 | 0.01, 0.11 | 0.02 |
| Linear Interaction term (Group • days) | -1.7 | -2.2, -1.2 | <0.0001 |
| Quadratic Time After PEX Onset (days^2^) | -0.08 | -0.12, -0.05 | <0.0001 |
| Linear Time After PEX Onset (days) | 2.1 | 1.7, 2.5 | <0.0001 |
| Difference in FEV1% at PEX Onset (NSR - SR) | -15.3 | -31.0, 0.4 | 0.06 |
| Constant (FEV1%) | 53.4 | 42.2, 64.6 | <0.0001 |
|  |  |  |  |

**Table S3**. Linear mixed model (LMM) describing FEV1% as a function of time between two PEX treatment response groups, sustained responders (SRs, n = 10) and non-sustained responders (NSRs, n = 10). C.I. = confidence interval. Number of observations = 62. Number of subjects = 20. Observations per group: minimum = 1, maximum = 7, average = 3.1.

| **Model Parameter** | **Coefficient (β)** | **95% C.I. (β)** | **p-value** |
| --- | --- | --- | --- |
|  |  |  |  |
| Linear Interaction term (Group • days) | 0.052 | -0.003, 0.108 | 0.06 |
| Quadratic Time After PEX Onset (days^2^) | 0.006 | 0.002, 0.011 | 0.007 |
| Linear Time After PEX Onset (days) | -0.14 | -0.19, -0.08 | <0.0001 |
| Difference in ln(Serum IL-6) at PEX Onset (NSR - SR) | -0.01 | -0.57, 0.54 | 0.96 |
| Constant [ln(Serum IL-6)] | 1.69 | 1.28, 2.10 | <0.0001 |
|  |  |  |  |

**Table S4**. Linear mixed model (LMM) describing ln(serum IL-6) over time between two PEX treatment response groups, sustained responders (SRs, n = 10) and non-sustained responders (NSRs, n = 10). C.I. = confidence interval. Number of observations = 98. Number of subjects = 20. Observations per group: minimum = 3, maximum = 8, average = 4.9.

| **Model Parameter** | **Coefficient (β)** | **95% C.I. (β)** | **p-value** |
| --- | --- | --- | --- |
|  |  |  |  |
| Linear Interaction term (Group • days) | 0.04 | -0.01, 0.09 | 0.09 |
| Quadratic Time After PEX Onset (days^2^) | 0.006 | 0.002, 0.010 | 0.007 |
| Linear Time After PEX Onset (days) | -0.13 | -0.18, -0.08 | <0.0001 |
| Difference in ln(Serum hepcidin-25) at PEX Onset (NSR - SR) | -0.24 | -0.75, 0.28 | 0.37 |
| Constant [ln(Serum hepcidin-25)] | 3.87 | 3.49, 4.25 | <0.0001 |
|  |  |  |  |

**Table S5**. Linear mixed model (LMM) describing ln(serum hepcidin-25) over time between two PEX treatment response groups, sustained responders (SRs, n = 10) and non-sustained responders (NSRs, n = 10). C.I. = confidence interval. Number of observations = 98. Number of subjects = 20. Observations per group: minimum = 3, maximum = 8, average = 4.9.

| **Model Parameter** | **Coefficient (β)** | **95% C.I. (β)** | **p-value** |
| --- | --- | --- | --- |
|  |  |  |  |
| Quadratic Interaction term (Group • days^2^) | -0.006 | -0.011, -0.0004 | 0.04 |
| Linear Interaction term (Group • days) | 0.02 | -0.02, 0.07 | 0.36 |
| Quadratic Time After PEX Onset (days^2^) | 0.001 | -0.026, 0.004 | 0.68 |
| Linear Time After PEX Onset (days) | 0.03 | 0.002, 0.066 | 0.04 |
| Difference in ln(Serum iron) at PEX Onset (NSR - SR) | 0.32 | -0.10, 0.75 | 0.14 |
| Constant [ln(Serum iron)] | 3.43 | 3.14, 3.73 | <0.0001 |
|  |  |  |  |

**Table S6**. Linear mixed model (LMM) describing ln(Serum iron) over time between two PEX treatment response groups, sustained responders (SRs, n = 10) and non-sustained responders (NSRs, n = 10). C.I. = confidence interval. Number of observations = 98. Number of subjects = 20. Observations per group: minimum = 3, maximum = 8, average = 4.9.

| **Model Parameter** | **Coefficient (β)** | **95% C.I. (β)** | **p-value** |
| --- | --- | --- | --- |
|  |  |  |  |
| Quadratic Interaction term (Group • days^2^) | 32 | 17, 48 | <0.0001 |
| Linear Interaction term (Group • days) | -132 | -264, 1 | 0.05 |
| Linear Time After PEX Onset (days) | -9 | -100, 82 | 0.85 |
| Quadratic Time After PEX Onset (days^2^) | -2 | -10, 7 | 0.72 |
| Difference in sputum iron at PEX Onset (NSR - SR) | -343 | -1,425, 739 | 0.53 |
| Constant (sputum iron) | 2200 | 1500, 3000 | <0.0001 |
|  |  |  |  |

**Table S7**. Linear mixed model (LMM) describing sputum iron (ng/mg of sputum) over time between two PEX treatment response groups, sustained responders (SRs, n = 10) and non-sustained responders (NSRs, n = 10). C.I. = confidence interval. Number of observations = 63. Number of subjects = 18. Observations per group: minimum = 1, maximum = 7, average = 3.5.

| **Parameter** | **Interpretation** |
| --- | --- |
|  |  |
| Hemoglobin, gm/dl | No between-cohort difference |
| Hematocrit, % | No between-cohort difference |
| RDWSD, fl | No between-cohort difference |
| RDWCV, % | Different time trends between cohorts |
| MCHC, gm/dl | Different time trends between cohorts |
| MCV, fl | Different time trends between cohorts |
| WBC, x10^3^/μl | No between-cohort difference |
| WBC Neutrophils, % | Borderline different time trends between cohorts |
| WBC Lymphocytes, % | Borderline different time trends between cohorts |
| WBC Monocytes, % | No between-cohort difference |
| WBC Basophils, % | Different time trends between cohorts |
| WBC Eosinophils, % | Different time trends between cohorts |
| Platelets, x10^3^/μl | Different time trends between cohorts |
| MPV, fl | Different time trends between cohorts |
| TSAT, % | Borderline different time trends between cohorts |
| TIBC, μg/dl | No between-cohort difference |
|  |  |

**Table S8**. Summary of findings about the trend differences between SRs and NSRs based on the final LMMs for all variables. Different time trends between cohorts means the quadratic and or linear interaction(s) between cohort and time was significant with p<0.05 while borderline means neither term is significant but the p-value was within 0.05-0.1. No between-cohort difference means neither interaction term was significant and the p-value>0.1. RDWSD = standard deviation of red cell distribution width; RDWCV = coefficient of variation of red cell distribution width; MCHC = mean corpuscular hemoglobin concentration; MCV = mean corpuscular volume; WBC = white blood cell; MPV = mean platelet volume; TSAT = transferrin saturation; TIBC = total iron binding capacity.

| **Predictor** | **Obs**  **(n)** | **Subjects**  **(n)** | **Coefficient of**  **Predictor** | **p-value**  **(Predictor)** | **Coefficient of CFRSD-CRISS**  **at PEX Onset** | **p-value**  **(CFRSD-CRISS**  **at PEX Onset)** |
| --- | --- | --- | --- | --- | --- | --- |
|  |  |  |  |  |  |  |
| ΔFEV1% | 35 | 12 | -0.70 | <0.001 | 0.60 | 0.01 |
| ΔHemoglobin, gm/dl | 75 | 20 | 0.99 | 0.48 | 0.49 | 0.02 |
| ΔHematocrit, % | 75 | 20 | 0.05 | 0.91 | 0.50 | 0.02 |
| ΔRDWSD, fl | 75 | 20 | -1.03 | 0.08 | 0.52 | 0.01 |
| ΔRDWCV, % | 75 | 20 | -1.22 | 0.43 | 0.51 | 0.01 |
| ΔMCHC, gm/dl | 75 | 20 | 2.59 | 0.14 | 0.54 | 0.01 |
| ΔMCV, fl | 75 | 20 | -1.50 | 0.047 | 0.46 | 0.04 |
| ΔWBC, x10^3^/μl | 75 | 20 | 0.29 | 0.36 | 0.50 | 0.02 |
| ΔWBC Neutrophils, % | 65 | 19 | 0.31 | <0.001 | 0.53 | 0.001 |
| ΔWBC Lymphocytes, % | 65 | 19 | -0.40 | 0.001 | 0.58 | 0.001 |
| ΔWBC Monocytes, % | 65 | 19 | 0.05 | 0.93 | 0.51 | 0.01 |
| ΔWBC Basophils, % | 65 | 19 | -0.69 | 0.85 | 0.52 | 0.02 |
| ΔWBC Eosinophils, % | 65 | 19 | -0.59 | 0.04 | 0.46 | 0.01 |
| ΔPlatelets, x10^3^/μl | 75 | 20 | -0.01 | 0.61 | 0.54 | 0.01 |
| ΔMPV, fl | 75 | 20 | 1.29 | 0.65 | 0.52 | 0.02 |
| ΔSerum IL-6, pg/ml | 76 | 20 | 0.23 | 0.005 | 0.66 | 0.004 |
| ΔSerum hepcidin-25, ng/ml | 76 | 20 | 0.03 | 0.02 | 0.53 | 0.02 |
| ΔSerum iron, μg/dl | 76 | 20 | -0.09 | 0.002 | 0.44 | 0.02 |
| ΔSputum iron, ng/mg | 42 | 12 | -0.0006 | 0.52 | 0.41 | 0.03 |
| ΔTSAT, % | 76 | 20 | -0.20 | 0.03 | 0.47 | 0.01 |
| ΔTIBC, μg/dl | 76 | 20 | -0.10 | <0.001 | 0.55 | 0.02 |
|  |  |  |  |  |  |  |

**Table S9**. LMMs for CFRSD-CRISS in response to changes in physiologic and/or biochemical indices, respectively, with control for CFRSD-CRISS at PEX onset. Each coefficient means the expected change in CFRSD-CRISS for every unit increase in the corresponding predictor variable. RDWSD = standard deviation of red cell distribution width; RDWCV = coefficient of variation of red cell distribution width; MCHC = mean corpuscular hemoglobin concentration; MCV = mean corpuscular volume; WBC = white blood cell; MPV = mean platelet volume; TSAT = transferrin saturation; TIBC = total iron binding capacity.

**
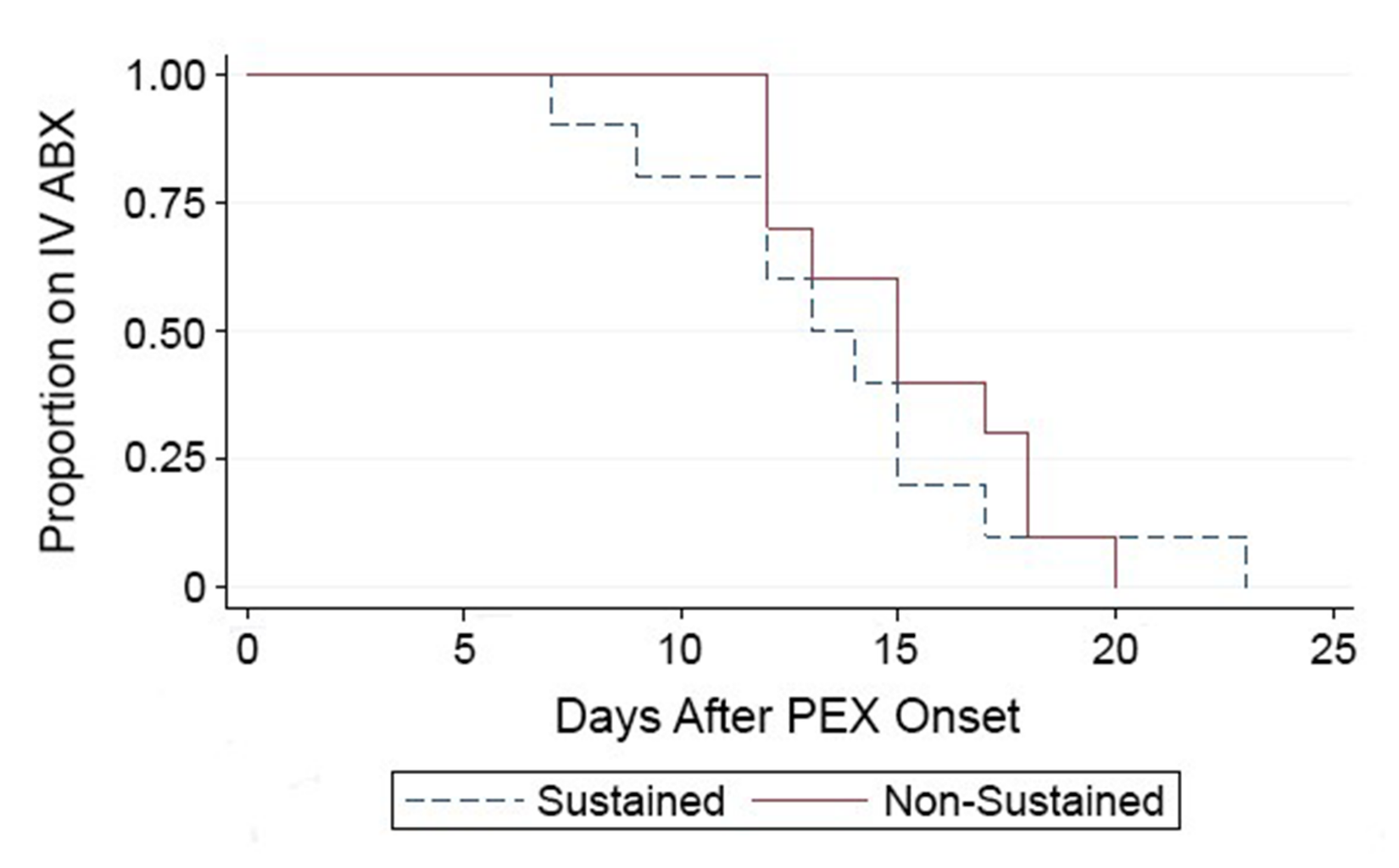
**

**Fig. S1**. Kaplan-Meier estimator curves depicting the length of PEX treatment with IV ABX for SRs and NSRs. The median length of treatment for SRs is 13.5 days and that for NSRs is 15 days. The Chi-square statistic for the log-rank test of equality of medians was 0.37 (p = 0.54).


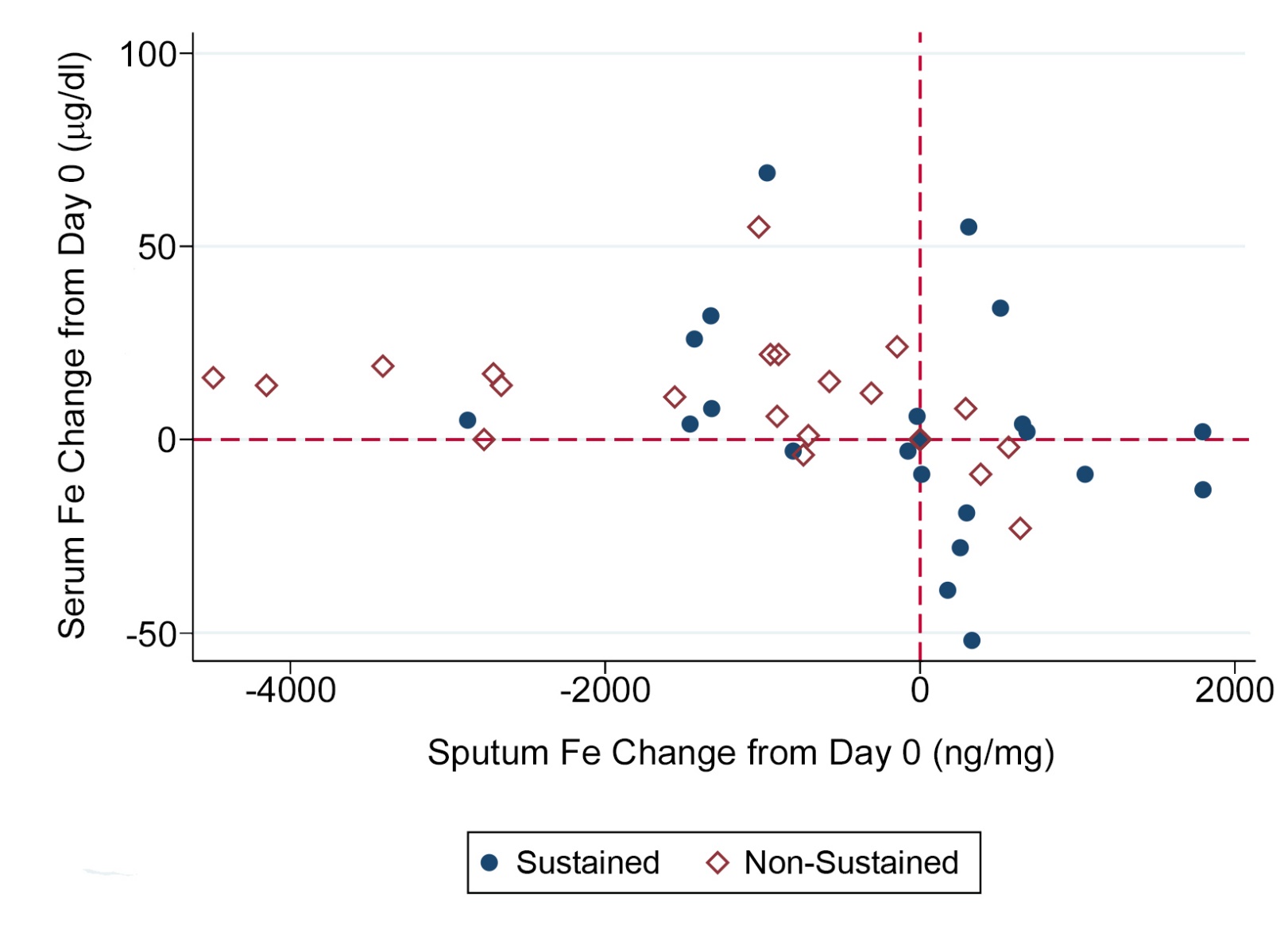


**Fig. S2**. Correlation between treatment-associated changes in serum and sputum iron over time beginning with treatment day 0. The correlation coefficient is -0.34 (p = 0.02).
